# HairNet2: deep learning to quantify cotton leaf hairiness, a complex genetic and environmental trait

**DOI:** 10.1186/s13007-024-01149-8

**Published:** 2024-03-19

**Authors:** Moshiur Farazi, Warren C. Conaty, Lucy Egan, Susan P. J. Thompson, Iain W. Wilson, Shiming Liu, Warwick N. Stiller, Lars Petersson, Vivien Rolland

**Affiliations:** 1 Data61, Commonwealth Scientific and Industrial Research Organisation, Clunies Ross street, Canberra, 2601 Australian Capital Territory Australia; 2 Australian Cotton Research Institute, 21888 Kamilaroi Hwy, Narrabi, 2390 New South Wales Australia; 3 Agriculture and Food, Commonwealth Scientific and Industrial Research Organisation, Clunnies Ross St, Canberra, 2601 Australian Capital Territory Australia

**Keywords:** Deep learning, Neural network, Machine learning, Phenotyping, Trichome, Cotton, Leaf, HairNet

## Abstract

**Background:**

Cotton accounts for 80% of the global natural fibre production. Its leaf hairiness affects insect resistance, fibre yield, and economic value. However, this phenotype is still qualitatively assessed by visually attributing a Genotype Hairiness Score (GHS) to a leaf/plant, or by using the HairNet deep-learning model which also outputs a GHS. Here, we introduce HairNet2, a quantitative deep-learning model which detects leaf hairs (trichomes) from images and outputs a segmentation mask and a Leaf Trichome Score (LTS).

**Results:**

Trichomes of 1250 images were annotated (AnnCoT) and a combination of six Feature Extractor modules and five Segmentation modules were tested alongside a range of loss functions and data augmentation techniques. HairNet2 was further validated on the dataset used to build HairNet (CotLeaf-1), a similar dataset collected in two subsequent seasons (CotLeaf-2), and a dataset collected on two genetically diverse populations (CotLeaf-X). The main findings of this study are that (1) leaf number, environment and image position did not significantly affect results, (2) although GHS and LTS mostly correlated for individual GHS classes, results at the genotype level revealed a strong LTS heterogeneity within a given GHS class, (3) LTS correlated strongly with expert scoring of individual images.

**Conclusions:**

HairNet2 is the first quantitative and scalable deep-learning model able to measure leaf hairiness. Results obtained with HairNet2 concur with the qualitative values used by breeders at both extremes of the scale (GHS 1-2, and 5-5+), but interestingly suggest a reordering of genotypes with intermediate values (GHS 3-4+). Finely ranking mild phenotypes is a difficult task for humans. In addition to providing assistance with this task, HairNet2 opens the door to selecting plants with specific leaf hairiness characteristics which may be associated with other beneficial traits to deliver better varieties.

## Background

### A need for robust and quantitative phenotyping tools to tackle complex crop traits

Understanding and exploiting beneficial or detrimental crop properties (phenotypes, or traits) requires accurate and preferably quantifiable phenotyping methods. This is particularly important when these properties are difficult to measure and complex (i.e. influenced by genetic and environmental factors). In cotton, some important properties are seedling emergence, canopy size and architecture, radiation use efficiency, disease and insect resistance, fibre quality and fibre yield [1]. For all of these, a number of phenotyping methods are available, whether they are manual, visual, mechanical or digital.

A visual or manual method has the advantage of being independent of complex or expensive equipment. However, manual methods can be time consuming, labor intensive, induce repetitive strain injury, and be biased by the observer. For some phenotypes, only manual methods are available, which often limits the scale at which such observations can be made. Mechanical approaches are particularly useful for fibre quality and fibre yield [1]. Interestingly, in recent years an increasing number of digital methods to capture detailed plant information have been developed, utilizing RGB cameras [2–8], hyperspectral sensors [9–11], thermal cameras [1, 12], and LiDAR-based sensors [13, 14]. These techniques offer unique advantages for characterizing various cotton plant phenotypes, can be non-destructive, and enable higher throughput applications.

However, adoption of such methods at scale has been relatively limited. This can be explained by factors such as cost (including the requirement for specialized equipment), speed, scalability, ease of implementation (e.g., access to code, super computers or graphical interface), or lack of demonstrated reproducibility in other experimental set-ups or commercial breeding programs. For example, whilst LiDAR-based techniques facilitate the measurement of plant height, canopy structure, and biomass by generating 3D representations of cotton plants, collecting such data requires robots [15] or rotary winged UAVs [16] which need to be manually or semi-autonomously navigated in the field for data collection. Uptake of a new method typically requires a significant improvement in speed, cost, reproducibility, scalability, or an ability to provide novel insights worthy of the extra time/money investment (e.g., a quantitative method rather than a qualitative one).

### Leaf hairiness is a key trait in Cotton

Leaf hairiness, also called pubescence, is a good example of an important and complex genetic trait in need of an improved phenotyping method. Leaf hairiness is determined by the amount, type and distribution of hair-like cells called trichomes on the abaxial side (underside) of leaves. In cotton, this phenotype has been shown to impact the ability of the plant to resist different types of insect pests. Leaves with no or few hairs are susceptible to boll weevil (*Anthonomus grandis*), cotton aphid (*Aphis gossypii*), Asiatic cottonworm (*Spodoptera littoralis*), spotted bollworm (*Earias fabia*), green leafhopper and jassids (*Empoasca spp*), pink bollworm (*Pectinophora gossypiella*), tobacco budworm (*Helicoverpa virescens*) and several Lygus species, whilst those with a lot of hairs tend to be susceptible to silverleaf whitefly (*Bemisia tabaci*) [17, 18]. Interestingly, leaf hairiness also impacts fibre yield because of a genetic relationship between the development of trichomes on the leaf and of fibres (modified trichomes) on the developing seed. Lines with glabrous leaves (ie. without hairs) tend to have a decreased yield potential [19]. Conversely, high leaf hairiness can also negatively affect economic fibre value as mechanical harvesting of such varieties increases gin trash (the accumulation of leaf matter, stalks and dirt in harvested material) which in turn downgrades fibre colour and increases the amount of cleaning required prior to ginning [20]. To maximise insect resistance and minimise a deleterious effect on fibre yield and value, cotton breeders tend to select plants with an intermediate level of leaf hairiness.

### Current qualitative phenotyping methods for Cotton leaf hairiness

In commercial breeding programs such as that of the Commonwealth Scientific and Industrial Research Organisation (CSIRO, for a review of the program refer to [1]) this phenotype has been qualitatively measured for the last 50 years by humans using a ‘look and feel’ method relying on the tactile perception of hairs and their reflection of sunlight [8]. Based on this approach, a leaf, a plant or a plot is assigned a score (Genotype Hairiness Score, GHS) on a non-linear scale ranging from 1 (glabrous) and 5+ (pilose) similarly to that of Bourland et al. [21]. This scale contains 7 intermediate scores, namely 2, 3, 3/4, 4, $4/4+$ and 5. Typically, the GHS of the first fully expanded leaf (Leaf 3 from the top of the plant) from 6 plants from the same plot is estimated in the field by an expert and a global score is assigned to said plot based on these observations. Generally, genotypes with a leaf hairiness score between 3 and $4+$ are selected for subsequent breeding steps. This method is qualitative, subjective and prone to inter- and intra-operator variability - even with highly trained human experts. Due to its reliance on sunlight reflection, this method is also not used on cloudy days.

To address this issue, Rolland and Farazi et al. [8] built HairNet, a deep learning model, that can mimic human experts scores with high accuracy and reproducibility from cotton leaf images. HairNet achieved an impressive accuracy of 89% per image and 95% per leaf on its associated dataset (CotLeaf-1, available at [22]). Although HairNet is a robust model, it is still only qualitative and the accuracy of its predictions is limited by the quality and reliability of the ground truth annotations provided by human experts (ie. the level of hairiness of each genotype). To address this limitation, there is a need for a tool to quantify leaf hairiness from images. Attempts have been made to quantify hairiness in *Arabidopsis thaliana*, Soybean (*Glycine max*), Spring Wheat (*Triticum aestivum*) [23–26]. However, these methods rely on specialised imaging techniques (e.g., 3D X-ray computed tomography, 3D confocal laser scanning microscopy), and/or require time consuming and destructive sample preparation. Additionally, none of these exploit recent advances in deep learning. Of note is a recent paper in Cotton (*Gossypium hirsutum*) which uses deep learning to detect trichomes on leaves [27]. However, this method only detects trichomes on a small part of the edge of a leaf and relies on the use of a black background which limits its value to understand leaf hairiness across a leaf and at scale.

### HairNet2, a quantitative phenotyping tool for Cotton leaf hairiness based on deep learning

**Fig. 1:**
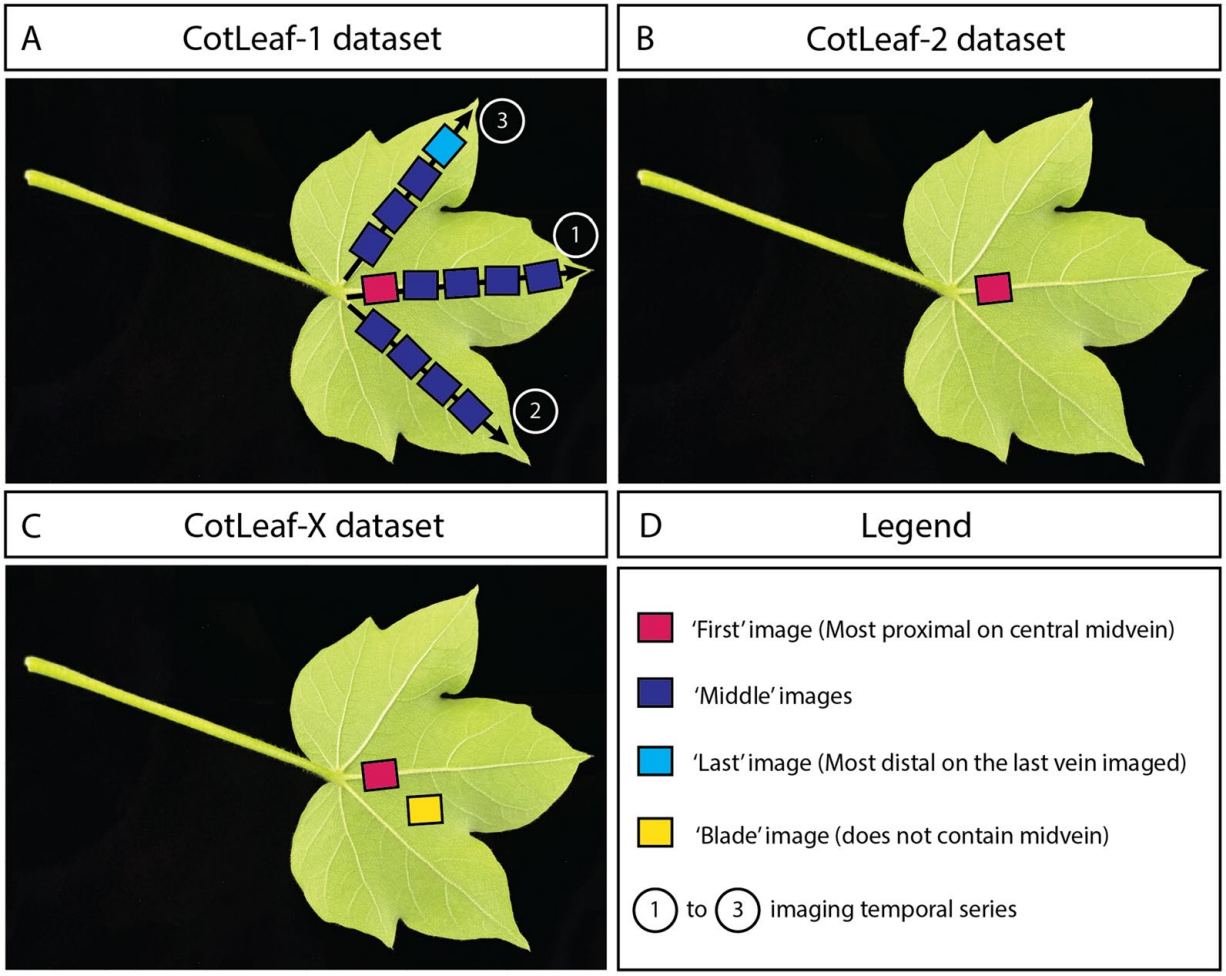
Description of the leaf locations at which images were taken in the CotLeaf-1 (**A**), CotLeaf-2 (**B**), and CotLeaf-X (**C**) datasets

**Fig. 2:**
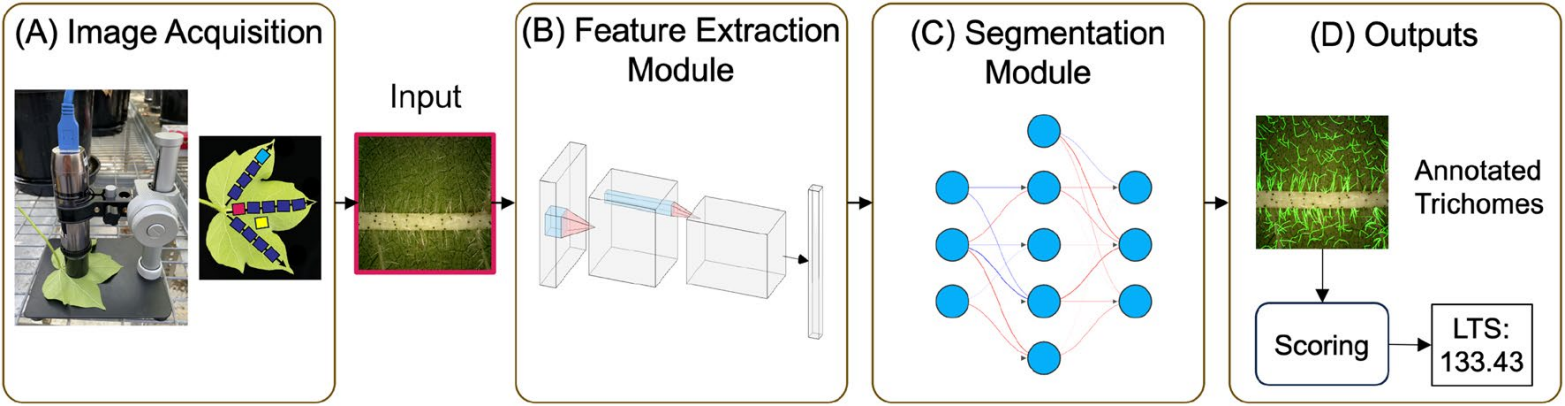
Architecture of the HairNet2 Model. Images were captured using the Image Acquisition (**A**) protocol described in [8]. The Feature Extraction Module (**B**) processes input images to derive essential visual features that capture the intricacies of leaf surfaces. The resulting distilled features are fed into the Segmentation Module (**C**), which differentiates trichomes from cotton leaf surface and produces a segmentation mask. The Outputs (**D**) consist of two parts: the predicted segmentation mask that highlights trichomes and a Leaf Trichome Score (LTS) quantifying the leaf hairiness and calculated from the segmentation mask

**Fig. 3:**
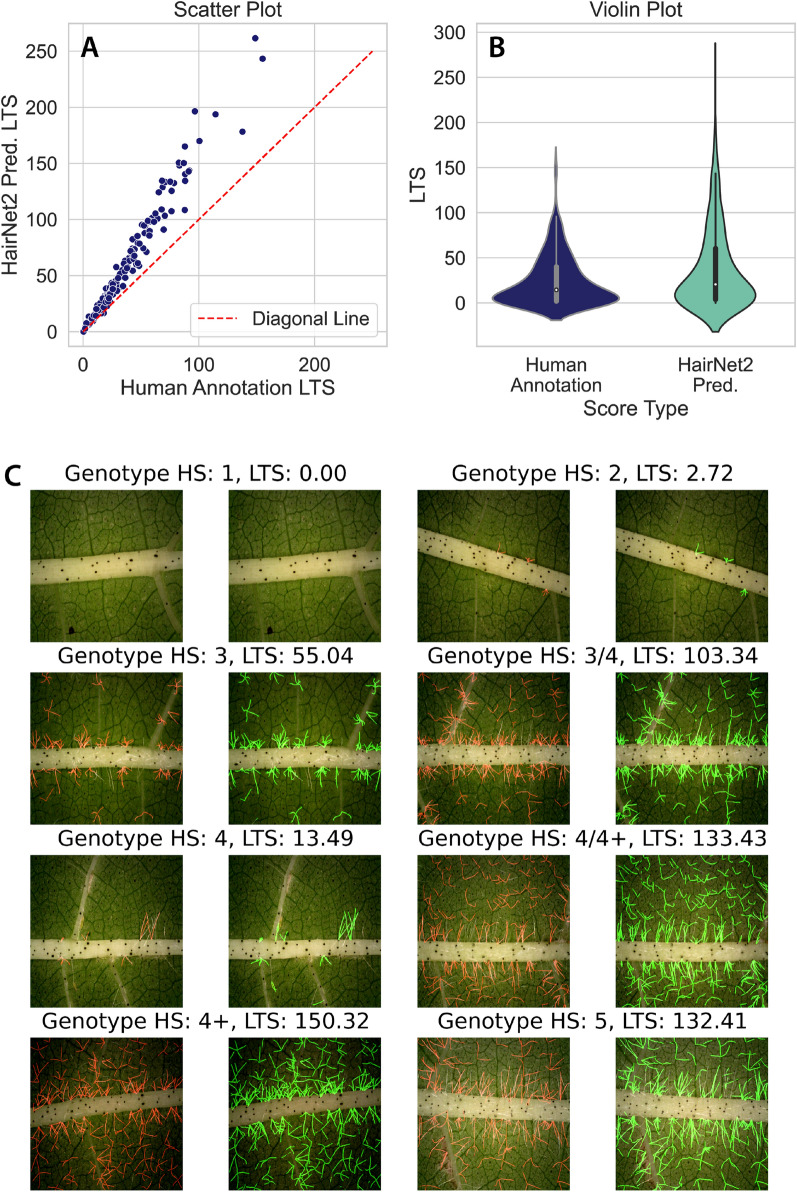
Comparison of LTS from human annotations with HairNet2 predictions on the 251 images of the AnnCoT Test split. The scatter plot (**A**) and the violin plot (**B**) show that HairNet2 tended to return higher LTS than human annotators. This was not due to the detection of false positives but more likely to the detection by HairNet2 of trichomes which had been missed by human annotators (**C**, red: human annotations, green: HairNet2 predictions). It is also possible that HairNet2 annotations were slightly wider than human annotations but this could not be quantified

**Fig. 4:**
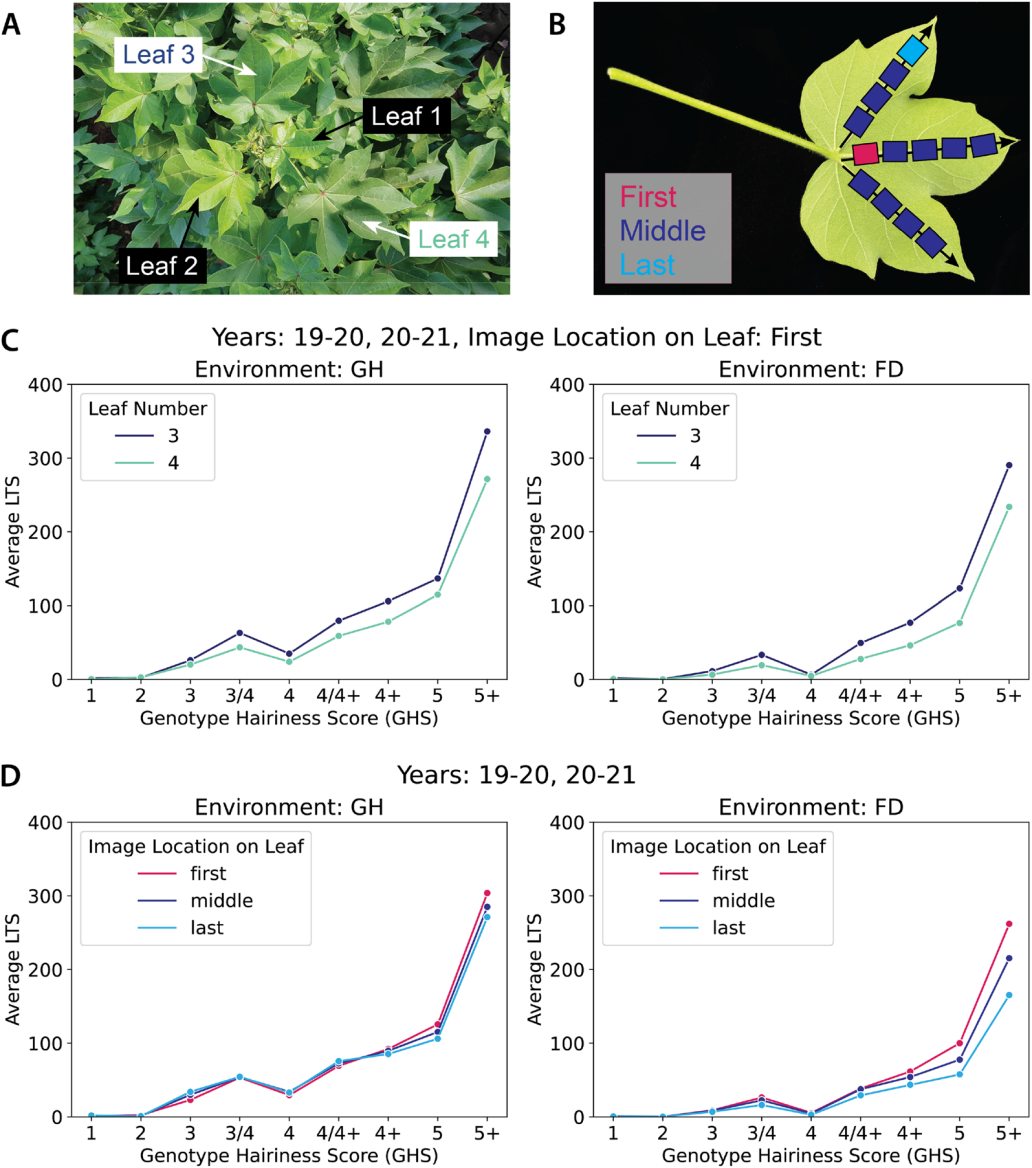
Effect of leaf number (**A**, **C**) and image location (**B**, **D**) on LTS predictions across GHS classes for both Glasshouse (GH) and Field (FD) conditions. These plots highlight that the overall trend of LTS increasing with GHS was not affected by leaf number or image position. However, a noticeable LTS dip was observed at GHS 4

**Fig. 5:**
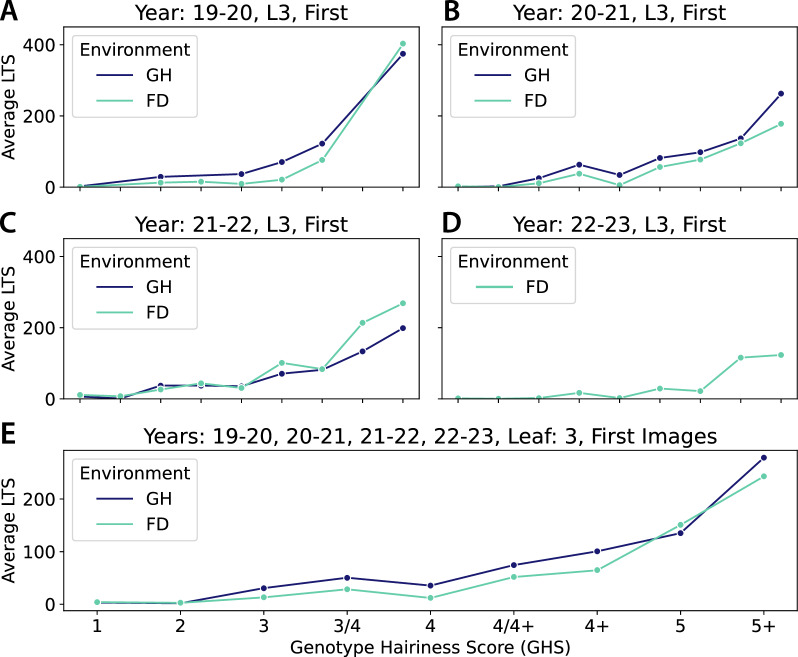
Effect of year to year variation on LTS across GHS classes in both Glasshouse (GH) and Field (FD) environments. Only L3 and First images were considered for this analysis. Whilst year to year variation was observed in terms of absolute LTS values, trends were similar for individual years (**A**–**D**) and all years combined (**E**). A dip in LTS at GHS 4 was observed on all years combined in both environments, with an additional dip at GHS 4+ detectable in some years as well. X axes on panels A-D are identical to the x axis in E

**Fig. 6:**
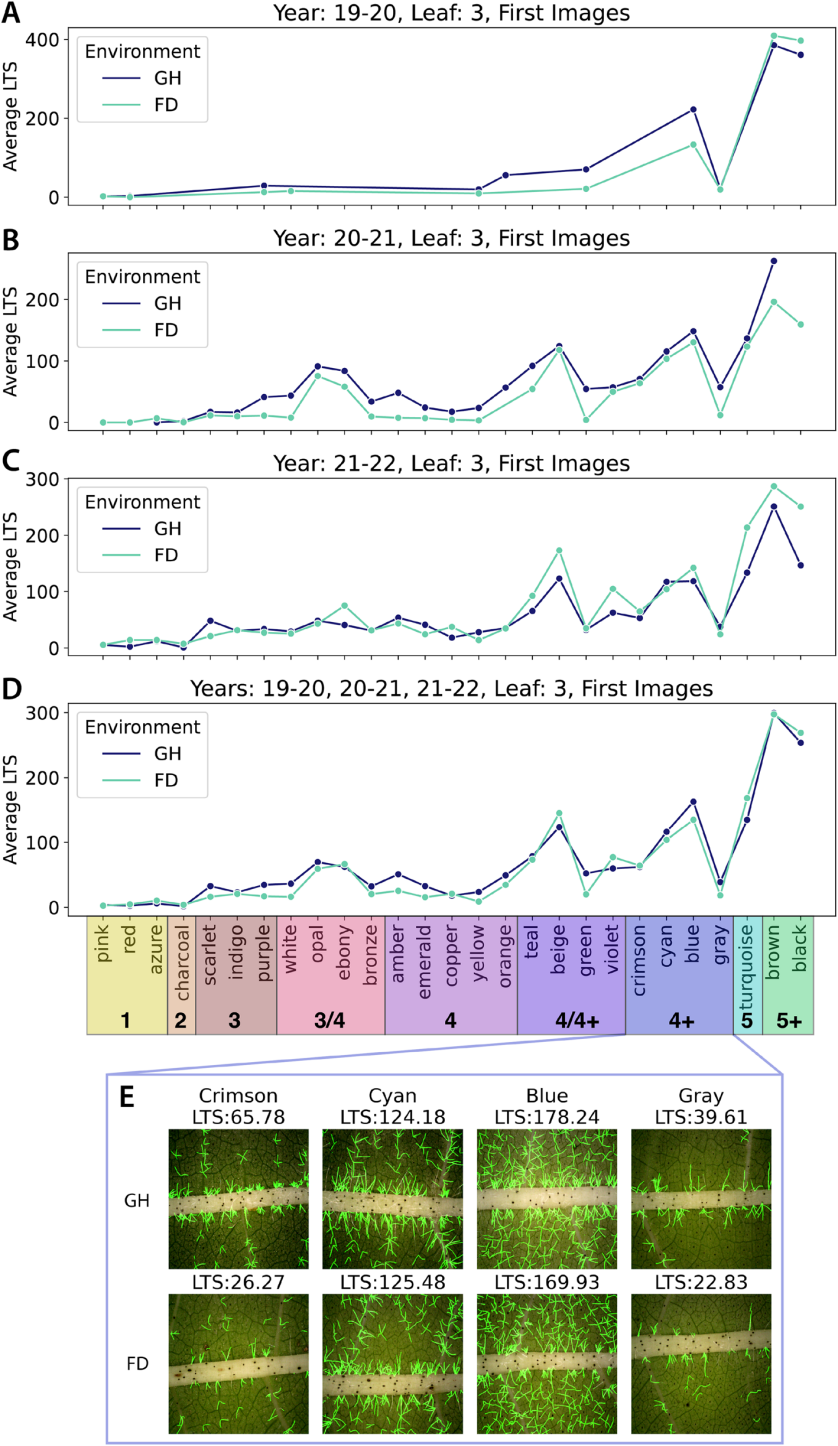
HairNet2 LTS predictions for individual genotypes across different years (**A**–**D**) and environments (GH: Glasshouse, FD: Field). GHS classes are colour coded on the x axis of panel D and qualitative examples of the four 4+ genotypes are shown in E to highlight the variations in LTS within single classes

**Fig. 7:**
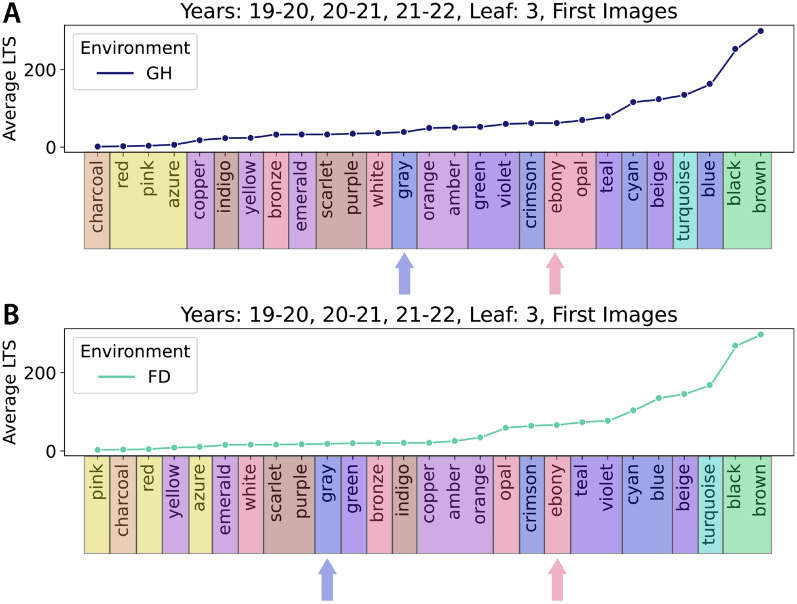
Proposed LTS-based genotypes ranking for the Glasshouse (GH, **A**) and the Field (FD, **B**). GHS classes are colour coded on the x axis of both panels and gray and ebony are highlighted with colours arrows to highlight the significant shift in their positions in the LTS-based ranking. This figure shows that LTS and GHS are in strong accordance for glabrous (GHS 1–2) and pilose (5–5+) genotypes but that genotypes with intermediate GHS are largely reorganised in the LTS-based rankings

**Fig. 8:**
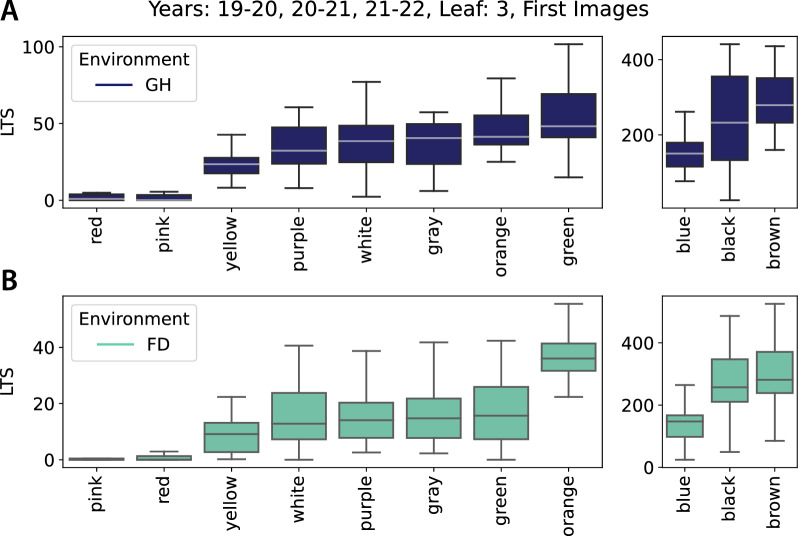
LTS distribution for the 10 genotypes common to Y1, Y2 and Y3 in the Glasshouse (GH, **A**) and the Field (FD, **B**). In each panel, the left plot displays less hairy genotypes, whilst the right plot shows the hairier ones which use a different y axis range

**Fig. 9:**
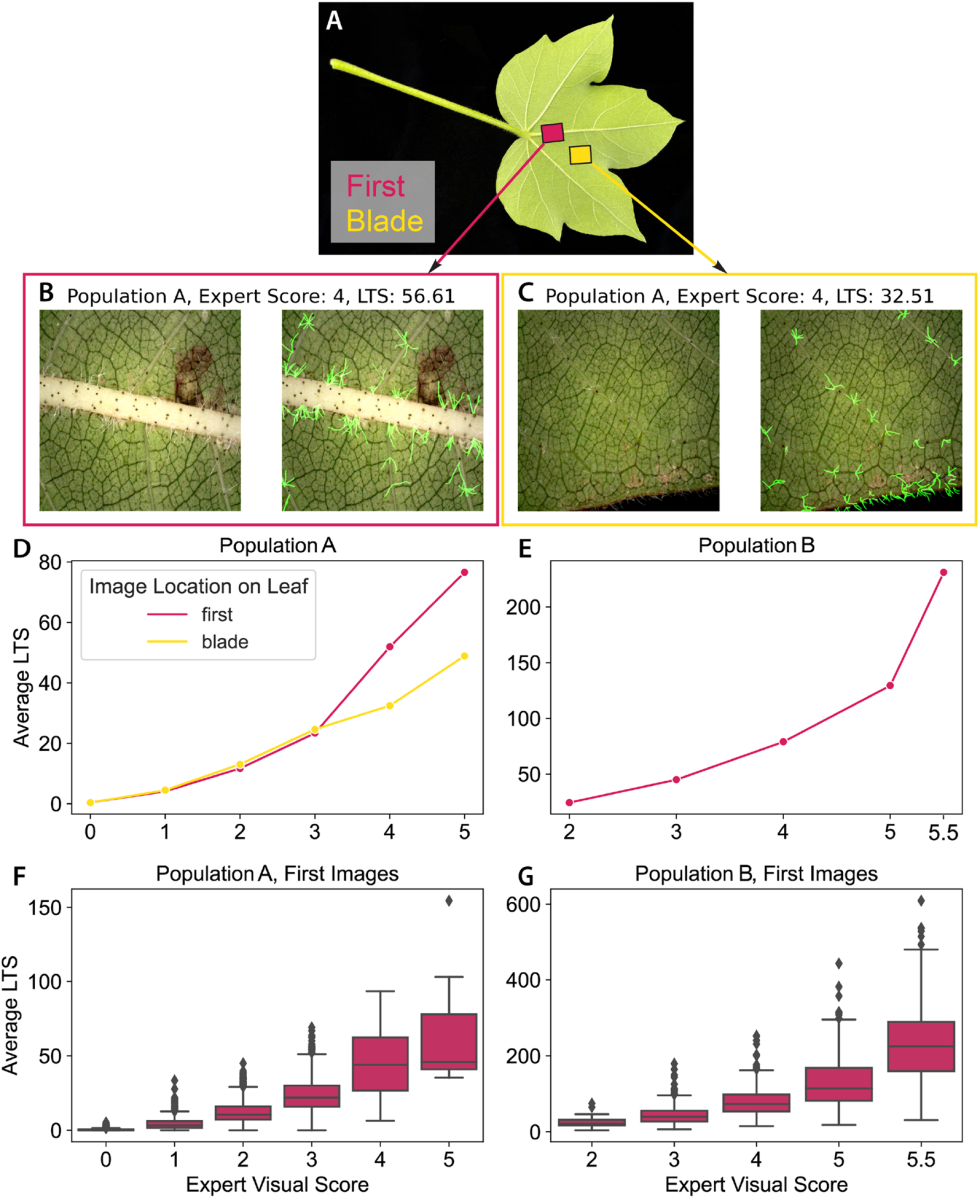
LTS from HairNet2 and expert visual image rankings on the CotLeaf-X dataset are highly correlated. CotLeaf-X includes First and Blade imaged from population A (**A**-**C**) and First images from population B (A). Plots in **D**–**G** show that for both populations the predicted LTS highly correlated with the visual scoring of images by experts

In this paper, it was hypothesized that leaf hairiness could be quantified from images using deep learning. To that end, the previously published CotLeaf-1 dataset (Fig. 1, Tables 1 and 3) was leveraged to create an annotated leaf trichome dataset called AnnCoT (Tables 1 and 4). AnnCoT was used to build a modular HairNet2 model composed of a feature extractor and a segmentation module (Fig. 2) to output a quantitative hairiness metric called Leaf Trichome Score (LTS) (Tables 5 and 6, 7 and Fig. 3). Two new image datasets were generated for this study (CotLeaf-2 and CotLeaf-X, Table 1) to compare LTS and GHS across leaves, growth environments and years (Figs. 4, 5 and 6). Based on the results of these experiments, a new LTS-based genotype ranking was proposed (Fig. 7). HairNet2 was further validated by analysing the distribution of its LTS values across years (Fig. 8) and by comparing its performance to that of human experts ranking images according to their hairiness (Fig. 9). To our knowledge, HairNet2 is the first quantitative tool to measure leaf hairiness at scale. It will enable accurate leaf hair phenotyping, which is central to both understanding the complex genetics underpinning this trait as well as untangling its effect on insect resistance and fibre yield. Ultimately this tool will be deployed in breeding programs to develop better cotton varieties.

## Methods

### **Datasets**

CotLeaf-1, CotLeaf-2 and CotLeaf-X datasets are summarized in Table 1 and Fig. 1 and details about their design, acquisition and composition are described below.

**Table 1:** Detailed Comparison of CotLeaf-1, CotLeaf-2 and CotLeaf-X Datasets. These three datasets differ in terms of season (Year, Y), growing environment (GH: Glasshouse, FD: Field), leaf number (L3 or L4), planting location, number of genotypes (see Table 2) or populations (A or B) imaged, total number of images, image location (see Fig. 1), presence/absence of multiple images per leaf, hairiness scale, and how hairiness scores were attributed

Characteristics	CotLeaf-1	CotLeaf-2	CotLeaf-X
	[8, 22]	(this study)	(this study)
Year (Y)	Y1, Y2	Y3, Y4	Y3
Environment	GH, FD	GH (Y3 only), FD	FD
Leaf number	3, 4	3	3
Location	Narrabri, Canberra	Narrabri	Narrabri
Num. of Genotypes (G) or Populations (P)	27 (G)	27 (G)	2 (P)
Num. of Images	13597	810	5049 (A:3276, B:1773)
Image Loc.	First, Middle, Last	First	First, Blade
Multiple images/leaf	Yes	No	Yes (Pop. A) No (Pop. B)
Genotype hairiness Scale (GHS)	{’1’, ’2’, ’3’, ’3/4’, ’4’, ’4/4+’, ’4+’, ’5’, ’5+’}	{’1’, ’2’, ’3’, ’3/4’, ’4’, ’4/4+’, ’4+’, ’5’, ’5+’}	(Pop. A) {’0’, ’1’, ’2’, ’3’, ’4’, ’5’}, (Pop. B) {’2’, ’3’, ’4’, ’5’, ’5.5’}
Score attributed by	Genotype	Genotype	Individual image

#### CotLeaf-1: Cotton Leaf Surface Images dataset 2019-21

CotLeaf-1 was published as part of [8] and is publicly available at [22]. It consists of 13,597 leaf surface images of $2560 \times 1920$ px, from 27 de-identified genotypes covering the full gamut of hairiness scores as shown in Table 2.

#### *CotLeaf-2:Cot*ton *Leafsurface images dataset 2021-23*

The Cot-Leaf-2 data set was collected from plants grown at Narrabri, NSW Australia under field (FD) and glasshouse (GH) conditions in 2021-22 (Y3) and only FD conditions in 2022-23 (Y4). The same suite of 27 genotypes in the Cot-Leaf-1 data set was studied.

##### Field experiments

Plants of the 27 genotypes were established in the summer growing seasons of 2021-22 and 2022-23 at the Australian Cotton Research Institute (ACRI, $-$30.21, 149.60), 22 km north-west of Narrabri New South Wales, Australia. Seeds of each genotype were planted on the 23rd of October 2021 (Y3) and the 19th of November 2022 (Y4), at planting density of 10–12 plants m^-2^ in rows spaced at 1 m. Each genotype was grown in a single 13 m row.

The study region is semi-arid, characterised by mild winters, hot summers and summer-dominant rainfall patterns. The region has an annual average precipitation of 646 mm [28]. The soil of the site is a uniform grey cracking clay (USDA soil taxonomy: Typic Haplustert; Australian soil taxonomy: Grey Vertosol). Plant available soil water to 1.2 m at the site is between 160 and 180 mm [29]. The soil at ACRI is generally 60–65 per cent clay fraction, of low drainage rate [30], pH range of 8.0–8.8, and low in organic matter and nitrogen [31].

Nitrogen was applied as urea approximately 12 weeks before planting at a rate of 240 kg N ha^-1^. Experiments were planted following an 11-month fallow period which was preceded by a winter wheat crop. Management for all field experiments followed current high-input commercial practices: fully irrigated conditions with careful weed and insect control [32]. Plants were furrow irrigated every 10–14 d (approximately 1 ML ha^-1^ applied at each irrigation) from December through to March, according to crop requirements. Each experiment was managed according to its individual requirements for irrigation and pest control, with all plots receiving the same management regime.

##### Glasshouse experiment

Plants were grown in temperature-controlled glasshouses at the Australian Cotton Research Institute (ACRI). About 15 seeds of each genotype were sown in 8 L plastic pots filled with soil on the 7th of November 2021. The soil was obtained from cotton fields at ACRI (see above). To improve the nutrient status of the potting mix 10 g of MULTIgro^®^ (Incitec Pivot Fertilizers, Melbourne, Australia) basal fertiliser was dissolved into the soil before planting. MULTIgro^®^ contains the nutrients N, P, K, S, and Ca at 13.1, 4.5, 7.2, 15.4, and 2.4 percent, respectively. A 10 mm layer of sand was added to the surface of the pots to reduce surface evaporation and assist in seedling emergence. Once emerged seedlings had reached the three-leaf stage, pots were thinned to three plants per pot. Plants were grown at 18 °C night and 32 °C during the day, under natural light conditions.

##### Leaf selection and imaging

During the 2021-22 season, leaf samples from these plants were collected on the 10th of January 2022 for the field experiment (at 11 weeks), and 11th of January 2022 for the glasshouse experiment (at 9 weeks). During season 2022-23, leaf samples from these plants were collected on the 23rd of January 2023 for the field experiment (at 9 weeks). For all these, Leaf 3 was harvested from 10 plants per genotype, placed in a paper bag and imaged the same day using the same protocol and equipment as in [8, 22]. Unlike in CotLeaf-1, for CotLeaf-2 only one image was collected per leaf, along the central midvein and corresponding to the ’first image’ in CotLeaf-1 as shown in Fig. 1. The abaxial side of leaves were imaged at a magnification of about 31x with a portable AM73915 Dino-lite Edge 3.0 (AnMo Electronics Corporation, Taiwan) microscope equipped with a RK-04F folding manual stage (AnMo Electronics Corporation, Taiwan) and connected to a digital tablet running DinoCapture 2.0 (AnMo Electronics Corporation, Taiwan). The exact angle of the mid-vein in each image was not fixed. However, either end of the mid-vein was always cut by the left and right borders of the field of view, and never by the top and bottom ones. This dataset comprises 810 images.

#### *CotLeaf-X:CottonLeafSurface Images dataset with eXpert labels*

##### Plant genotypes and growth conditions

Two cotton populations called A and B, were selected for their heterogeneous leaf hairiness, with population A being generally less hairy than population B. Both populations were planted in the summer growing season of 2021-22 at ACRI. Seeds of each genotype were planted in a field on the 23rd of October 2021 at a planting density of 10–12 plants/m2 in rows spaced at 1 m. Each genotype was grown in a single 13 m plot.

##### Leaf selection and imaging

Leaf samples from these plant populations were collected on the 2nd and 6th of March 2022 (at 19 weeks, first open boll stage). Leaf 3 was harvested from 10 plants per genotype. Imaging was performed as described above with the following distinctions (Fig. 1C):

- for population A, two images were collected per leaf: one along the central midvein as in CotLeaf-2, and one on the leaf blade.
- for population B, one image was collected per leaf: along the central midvein as in CotLeaf-2. This dataset comprises 3276 images for population A and 1773 for population B.

##### Visual scoring of images by human expert

A human expert scored all CotLeaf-X images using arbitrary ordinal scales ($0-5$ for population A and $2-5.5$ for population B), where higher numbers corresponded to images with more trichomes.

**Table 2:** De-identified genotypes and their associated genotype hairiness scores (GHS)

GHS	De-identified genotype
1	’pink’, ’red’, ’azure’
2	’charcoal’
3	’scarlet’, ’indigo’, ’purple’
3/4	’white’, ’opal’, ’ebony’, ’bronze’
4	’amber’, ’emerald’, ’copper’, ’yellow’, ’orange’
4/4+	’teal’, ’beige’, ’green’, ’violet’
4+	’crimson’, ’cyan’, ’blue’, ’gray’
5	’turquoise’
5+	’brown’, ’black’

#### *AnnCoT:AnnotatedCottonTrichome dataset*

##### Image selection

A subset of the CotLeaf-1 dataset ([22]) was used to develop the Annotated Cotton Trichome (AnnCoT) dataset. Specifically, the first image Fig. 9 of Leaf 3 and Leaf 4 from each genotypes with hairiness scores between 1 and 5 grown in both environments (Glasshouse and Field) during Year 1 (season 2019-20) and Year 2 (season 2020-21) were used. Images from genotypes with a $5+$ genotype hairiness score were not annotated because they were too hairy for humans to confidently annotate. As a result, a total of 1250 images were annotated and their distribution across genotypes and hairiness classes is shown in Table 3

##### *Trichome spline annotation*

The above-mentioned images were annotated by humans tasked to trace each trichome with the exception of those only fully overlapping with the midvein. This decision was made because it was difficult to distinguish white trichomes on the white midvein from light reflection or damage. Annotations were stored as splines instead of pixel level segmentation masks at the original image resolution. This allowed for a more flexible and continuous representation of trichomes and provided two important methodological advantages. Firstly, spline annotations were stored as a set of control points manually placed by annotators on each trichome and processed into segmentation masks when needed, which was less expensive than storing segmentation masks themselves. Secondly, resizing a segmentation mask of thin hair-line structure such as trichomes would have introduced serious artefacts (e.g., breaking up long annotations, or merging nearby annotations into a single structure) due to interposition techniques (e.g., linear, cubic, nearest, area). Spline annotations offer the advantages of being ‘transformable’ to any image resolutions by calculating new control points for the target image resolution. The transformation operation is inexpensive and ensures that the original shape is preserved regardless of the resizing dimensions.

Through this process, 1250 images and their associated trichomes annotated as splines were used to generate the Annotated Cotton Trichome (AnnCoT) dataset. To develop HairNet2, the AnnCoT dataset was split into train, validation and test as shown in Table 4.

**Table 3:** Detail of the number of genotypes and images for each hairiness class in the AnnCoT dataset. GHS: Genotype hairiness score

GHS	# Images	# Genotypes
1	152	3
2	40	1
3	160	3
3/4	180	4
4	238	5
4/4+	200	4
4+	240	4
5	40	1
5+	0	0
**Total**	**1250**	**25**

**Table 4:** Size of the AnnCoT dataset training, validation, and test splits

Dataset	# Images
Train (65%)	812
Val (15%)	187
Test (20%)	251
**Total**	**1250**

### Problem formulation and model development

The task of trichome quantification from cotton leaf images was decomposed into two distinct components, segmentation and quantification, each addressing a specific aspect of the problem.

#### *Trichome segmentation*

The first challenge was to accurately identify and delineate trichomes present in cotton leaf images. This was formulated as a binary image segmentation task aiming to partition an input image $I$ into two distinct regions: trichomes (foreground) ($F$) and non-trichomes (background) ($B$). Let $M$ represent the binary segmentation mask, which assigns a binary label $m_{xy} \in \{0,1\}$ to each pixel coordinate $(x,y)$ in the image. Here, $m_{xy} = 1$ indicates that the corresponding pixel belongs to the foreground region, while $m_{xy} = 0$ indicates the background region. The objective was to estimate the optimal binary segmentation mask $M^*$ that accurately captured the foreground and background regions in the input image. The binary image segmentation problem can be mathematically expressed as:

1 $$\begin{aligned} M^* = \arg \min _M E(M) \end{aligned}$$

given that $m_{xy} \in \{0,1\}$ for all $(x,y)$ in the image.

While the segmentation mask shares the same dimensions as its corresponding leaf image, it is considerably sparser due to the vast difference between the number of trichome pixels and background pixels. Storing these masks in their uncompressed form would be an inefficient use of storage space. To optimize this, a protocol was adopted that stored only the row and column indices of non-zero values, which represent trichome pixels. This method greatly minimized the storage requirements for the segmentation masks and facilitated faster loading and processing of the segmentation data for subsequent analyses.

#### *Quantification of segmented trichomes*

To quantify segmented trichomes, a metric termed *Leaf Trichome Score (LTS)* was introduced. LTS was calculated based on the ratio of pixels segmented as trichomes over the total number of pixels in the image. Given the sparse nature of trichomes in most images, a scaling factor of $1000$ was introduced to make the score more interpretable and to scale it to a meaningful range for comparison. Mathematically, it can be expressed as:

2 $$\begin{aligned} \text {LTS} = \frac{N_{{\mathcal {S}}} \times 1000}{N_{{\mathcal {T}}}} \end{aligned}$$

with $N_{{\mathcal {S}}}$ denoting the number of pixels segmented as trichomes and $N_{{\mathcal {T}}}$ represents the total number of pixels in the image. The higher the LTS, the higher the number of trichome pixels in an image.

### Model architecture

#### *Feature extractor module*

Feature extractors from the following family of models were tested in this study:

- *VGG* [33] is one of the legacy deep CNNs that remains relevant due to its simplicity and effectiveness in image classification tasks. Various depths of VGG were explored, and VGG19 with batch normalization was used for experimentation in the feature extractor module.
- *ResNet* [34] introduced the concept of skip connections to deal with the vanishing gradient problem and allowed neural networks to substantially increase depth. It is one of the most widely used feature extractors in production because of its robustness and scalability, with varying depth offerings. ResNet18 and ResNet50 were tested in this study.
- *SENet* [35] utilizes Squeeze-and-Excitation (SE) blocks that perform adaptive re-calibration of channel-wise features, allowing the model to weigh spatial features based on the channel descriptors. This imparts a form of channel-wise attention on the model. SE-ResNet50 (ResNet50 fused with an SE block to improve performance) was tested in this study.
- *RegNet* [36] models offer uniform structures across networks, resulting in simple and regularized designs. Such networks are developed via automated exploration of the architecture design space. RegNetX64 was used in this study.
- *EfficientNet* [37] uses compound scaling of network depth, width, and resolution, and employs depth-wise separable convolutions. EfficientNet-B5 variant was used in this study.

#### *Segmentation module*

The following segmentation models were experimented with in this study:

- *UNet* [38] is a widely-used segmentation architecture known for its symmetrical U shaped encoder-decoder structure with skip connections that assist in retaining spatial details. The architecture was originally designed for biomedical image segmentation, however it has been quite successful in domain-specific applications where general purpose segmentation models like Mask-RCNN [39], YOLO [40] have been sub-optimal.
- *UNet++* [41] is an advanced variant of UNet that introduces nested and skip connections, providing a series of segmentation maps of different depths. This design improves the ability of the model to segment objects of various shapes and scales, which is relevant when looking at thin hair-like structures as in this study.
- *DeepLabv3* [42] introduces atrous convolutions with up-sampled filters and a ’Multilevel Atrous Spatial Pyramid Pooling’ (ASPP) operation. This approach has proven effective in capturing multi-scale context by utilizing multiple dilation rates and providing a more comprehensive contextual understanding. It is relevant in the context of trichome segmentation.
- *LinkNet* [43] is an efficient and lightweight architecture designed for semantic segmentation tasks. It employs an encoder-decoder structure where the encoder is based on a pre-trained classification network. The decoder integrates skip connections to combine low-level and high-level feature maps, resulting in a detailed segmentation map.
- *Multi-path Aggregation Network (MANet)* [44] focuses on effectively capturing multi-scale features by employing multi-path aggregations. By adjusting the receptive field, the model demonstrates robust performance across physical scales.

### Data augmentation

The following data augmentation techniques were employed in this study:

- *Resizing:* to reduce memory constraints and ensure images had the dimensions required to be fed to the feature extraction module, they were resized to lower dimensions, typically $512 \times 512$ pixels. It is important to note that segmentation masks were directly drawn at the resized image dimension, rather then the original size, to avoid undesirable artifacts and discontinuity in the masks.
- *Random Flip:* this operation involved a combined random vertical and horizontal flip, ensuring that the model would be invariant to the orientation of leaf patterns.
- *Random Crop:* this step involved cropping random sections of each image at smaller resolutions than the original image. This was performed to teach the model to recognize trichomes regardless of their position and scale in an image. The corresponding masks were identically cropped to maintain consistency.

### Loss functions

In this study, the following loss functions were tested:

- *Binary Cross-Entropy (BCE) Loss*. This loss is one of the default choices for binary image segmentation tasks. It is an intuitive fit as each pixel of the segmentation map is treated as an individual binary classification instance.
- *Dice Loss*. This loss is derived from the Sørensen-Dice coefficient, and it is also a common choice for binary and multi-class segmentation tasks. It is particularly relevant here because trichomes pixels were expected to be less common than background pixels and this loss function deals well with class imbalances as it places emphasis on the accurate classification of minority classes.
- *Jaccard (IoU) Loss*. This loss is also useful for its robustness against class imbalances as it measures the similarity between predicted and ground truth segmentation masks, making it particularly relevant for the problem at hand in this study.
- *Focal Loss*. This loss has also proven effective to address class imbalances in object detection. By down-weighting well-classified examples, it forces the model to focus on challenging image regions which is essential for detecting and segmenting intricate structures such as trichomes.

### Accuracy metrics for model evaluation

Intersection over Union (IoU) and F1-score were used to evaluate the segmentation performance of our HairNet2 model.

#### *Intersection over Union (IoU)*

IoU was defined as the ratio of the intersection area between the predicted segmentation mask $P$ and the ground truth mask $G$, over the union area of the two masks. Mathematically, this can be expressed as

3 $$\begin{aligned} IoU = \frac{{Area(P \cap G)}}{{Area(P \cup G)}} \end{aligned}$$

#### *F1-Score*

The $F_1$ score was used to evaluate segmentation performance. F1 provides a balance between precision and recall, offering a comprehensive insight into the accuracy of the segmentation. The $F_1$ score is given by:

4 $$\begin{aligned} F_1 = \frac{2 \times (\text {precision} \times \text {recall})}{(\text {precision} + \text {recall})} \end{aligned}$$

Where the precision and recall are defined as: $\text {precision} = |P \cap G| / |P| \quad \text {and} \quad \text {recall} = |P \cap G| / |G|$ with $P$ being the predicted segmentation mask and $G$ the ground truth mask.

## Results and discussion

### Using the CotLeaf-1 and AnnCoT datasets to build HairNet2

This study made use of the publicly available CotLeaf-1 dataset which served to build the qualitative leaf hairiness classification model HairNet ([8, 22]). This dataset consists of 13,597 cotton leaf images collected over 2 seasons: 2019-20 (also referred to as year 1 or Y1) and 2020-21 (year 2 or Y2). In Y1, 10 genotypes were grown in the field and in the glasshouse with 9 genotypes common between the two environments. In Y2 this was expanded to 27 genotypes grown in the field and glasshouse. During these seasons multiple images were taken on two leaves per plant (Leaf 3, also called L3, and Leaf 4, or L4). For details on this dataset refer to Material and Methods as well as Fig. 9, Table 1 and Rolland et al [8, 22].

A subset of 1250 images of CotLeaf-1 representing all Genotype Hairiness Scores (GHS) with the exception of 5+ were annotated by humans to provide ground-truth of trichomes on leaf surfaces (Table 3). This dataset was used to train the various HairNet2 architectures using the ’Train/Validation/Test’ splits shown in Table 4.

### Selecting the optimal HairNet2 architecture

HairNet2 was build around two key modules: a feature extractor and a segmentation module Fig. 2.

The purpose of the feature extractor network was to extract visual and salient features from the input (leaf images). It learns to capture both low-level visual details, such as edges and textures, and high-level semantic information, enabling it to produce meaningful representations of leaf structures. In this study, 6 pre-trained CNN-based feature extractors were tested, namely VGG19-bn, ResNet18, ResNet50, EffNet-B5, RegNetX-064 and SE-ResNet50 [33–37]. These models were pre-trained on the ImageNet dataset [45], a very large-scale dataset specifically designed for image classification task. Using pre-trained models for the feature extractor module presented two significant benefits. Firstly, pre-trained weights provided a robust foundational knowledge, improving the model’s ability to generalize from limited data as is the case here. Secondly, the model was able to extract more meaningful features and improve the model’s ability to segment very thin (single or a couple of pixels wide) hair-like structures. The pre-trained models were then optimized for our dataset to exploit their ability to extract hierarchical features from complex visual data.

Image features were then fed to a segmentation module. This module used encoded feature maps generated by the encoder together with the ground-truth segmentation mask to produce a pixel-wise segmentation mask for an leaf image by classifying each pixel into ’trichome’ and ’background’. The segmentation module consisted of an encoder pathway that captured the context and spatial information through down-sampling operations, followed by a decoder pathway that utilized skip connections to recover spatial details. The segmentation models tested in this study were Unet [38], Unet++ [41], DeepLabv3 [42], MANet [44] and LinkNet [43]. The segmentation module is trained from scratch for our proposed AnnCoT dataset.

#### ***Selecting the best feature extractor and segmentation module pair***

The modular nature of HairNet2 meant that various combinations of feature extractors and segmentation modules needed to be tested to identify the optimal network architecture. Intersection over Union (IoU) and F1 scores provide valuable insights into the overall performance of binary segmentation algorithms and were used as evaluation metrics. As shown in Table 5, of the 30 combinations tested the best performance was achieved with the VGG19-bn feature extractor combined with either the MANet (IoU: $0.6303$, F1 score: $0.7586$) or the LinkNet (IoU: $0.6268$, F1 score: $0.7545$) segmentation modules. The fact that VGG19-bn performed better than deeper or more complex feature extractors may be explained by two factors. Firstly, a simple architecture like VGG19-bn may have been more effective at capturing texture and fine details, which is crucial for segmenting thin structures like trichomes. Secondly, batch normalization may have helped stabilise the learning process to better generalise, which is critical when the dataset is not very large. The VGG19-MANet and CGG19-LinkNet combinations were therefore selected for further analysis.

**Table 5:**
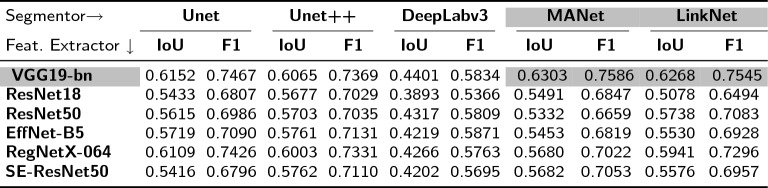
Performance evaluation for different feature extractor and segmentation module combinations. The table displays Intersection over Union (IoU) and F1 scores for various module pairs to identify the best performing pairs for trichome segmentation. Cells in grey value represent the tow top-performing combinations

#### ***The effect of data augmentation on selected module pairs***

**Table 6:**
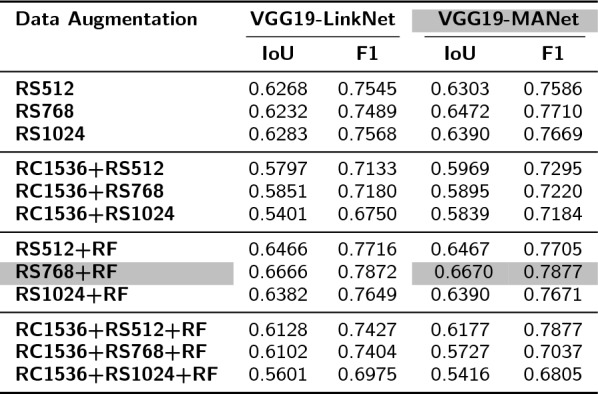
Comparison of the effect of various data augmentation techniques on the performance of the top two module pairs. Data augmentation methods tested here are: image resizing (RS) to 512 x 512 px (RS512), 768 x 768 px (RS768), 1024 x 1024 px (RS1024); random cropping (RC) to 1536 x 1536 px; random flip (RF). The best performing model + data augmentation pair is highlighted in grey value

To improve the generalization capabilities of HairNet2, reduce over-fitting and improve inference robustness, different types of data augmentation such as resizing, cropping and flipping were employed (Table 6).

When using resizing (RS) alone, VGG19-MANet with a resizing to $768 \times 768$ px produced the highest performance (IoU: $0.6472$, F1 score: $0.7710$) over smaller (512px) or higher (1024px) resizing strategies. Interestingly, when resizing was combined with random cropping (RC1536), model performance declined slightly, suggesting that aggressive cropping followed by resizing may have eliminated or distorted some crucial information. The addition of random flipping (RF) to resizing, especially RS768+RF, showed the best results with VGG19-MANet (IoU: $0.6670$, F1 score: $0.7877$) and confirmed the value of introducing beneficial variability to the dataset. When random cropping, resizing, and flipping were combined, a decline in model performance was evident across all models, with a significant drop in VGG19-MANet with ‘RC1536+RS1024+RF’. This highlighted that while individual or dual augmentation techniques can benefit the model, combining multiple data augmentation strategies introduced excessive variability in the data distribution, which did not translate in better generalization. While data augmentation generally enhances model robustness, it is essential to strike a balance to avoid over-complicating the input data. In the case of HairNet2, the highest performance was obtained with VGG19-MANet and a combination of resizing to $768 \times 768$ px followed by random flipping (‘RS768+RF’), with an IoU of $0.6670$ and a F1 score of $0.7877$.

#### ***Optimizing HairNet2***

**Table 7:**
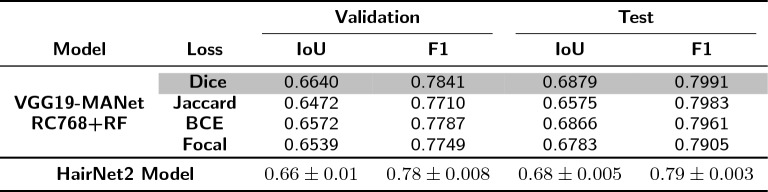
HairNet2 model accuracy on the Validation and Test splits of the AnnCoT dataset. These results were obtained by running the model 5 times, with the values denoted as ’mean ± range’

Given the nuances associated with segmenting intricate hair-like structures, and to further facilitate generalization of HairNet2 it was important to select an optimal loss function. The VGG19-MANet model with ’RC768+RF’ augmentation was subjected to four distinct loss functions: Dice, Jaccard, BCE, and Focal. The Dice loss performed better than all others loss functions tested with an IoU of $0.6640$ and a F1 score of $0.7841$ on the Validation set, and an IoU of $0.6879$ and a F1 score of $0.7991$ on the Test set.

This VGG19-MANet architecture with ’RC768+RF’ data augmentation and Dice loss function was therefore selected as the optimal model design, with the resulting model adopted as the final HairNet2. When run 5 times HairNet2 showed consistent performance, with a mean IoU of $0.66 \pm 0.01$ and a F1 score of $0.78 \pm 0.008$ on the Validation set. Similarly, for the test set the mean IoU and F1 scores were $0.68 \pm 0.005$ and $0.79 \pm 0.003$, respectively. This consistency, denoted by the narrow range of results, underscores the reliability of the HairNet2 model.

### HairNet2 efficiently detects leaf trichomes

The output of HairNet2 is a Leaf Trichome Score (LTS) which captures the quantity of pixels classified as belonging to trichomes in a given image (for details, refer to Material and Methods). Given that image size was constant, an increasing LTS corresponds to a more hairy leaf surface. Although IoU and F1 scores are standard metrics to evaluate the performance of segmentation tasks, in this instance it has limitations. This is because of the nature of trichomes, which are long and thin structures. For example, if HairNet2 was able to segment 10 trichomes of the right length in an image that indeed contained 10 trichomes but was misplacing them on the image, the IoU could be zero whilst the LTS would be accurate. Another example on the same image could be a situation where HairNet2 was able to segment the 10 trichomes properly according to location and length but was consistently doubling their width, the IoU would also be affected whilst the effect on LTS would be consistent across the same dataset, thereby not being problematic.

For these reasons, it was important to quantitatively and qualitatively interpret the performance of HairNet2 on the AnnCoT human annotations (ie human LTS) (Fig. 3). Quantitatively, HairNet was found to report slightly higher LTS values than human annotators generated (Fig. 3A and B). This could be due to a number of factors including the generation of false positives (ie. trichomes detected by HairNet2 which did not exist), segmentation of longer trichomes (e.g., correct number of trichomes but length overestimated), segmentation of thicker trichomes (e.g., correct number of trichomes but thickness overevaluated), or the detection of trichomes which had been missed by human annotators. Close qualitative inspection of a range of images showed than HairNet2 did not significantly generate false positives and did not overevaluate trichome length (Fig. 3C). Whilst a small increase in segmented trichome width was hard to assess in this case it is a possibility. However, it was evident that a number of trichomes that HairNet2 was able to pick up had been missed by human annotators (Fig. 3C) which is at least a partial explanation for the slightly higher LTS values observed with HairNet2 than with humans.

Overall, these results highlight the high level of accuracy of HairNet2, both in terms of LTS and its ability to find the vast majority of trichomes of the right length and in the right location.

### Analysis of LTS variations across leaves, image positions and years

Having demonstrated the strong ability of HairNet2 to segment leaf trichomes opened the door to revisit the CotLeaf-1 dataset for which images had been classified by HairNet based on the Genotype Hairiness Score (GHS) provided by breeders. In Rolland et al. ( [8]), the authors had demonstrated that the GHS classification performance of HairNet was largely independent of leaf identity (L3 vs L4, Fig. 4A), or the number/position of the images used for classification (First vs All in HairNet, further subdivided here into First, Middle, and Last; Fig. 4B).

In both L3/L4 and First/Middle/Last comparisons, LTS values increased with GHS with the notable exception of a notable dip at GHS 4 (Fig. 4C and D). Whilst the absolute values between Field and Glasshouse were slightly different, within a given environment both L3 and L4 exhibited very similar patterns, noting that L4 returned slightly lower LTS values (Fig. 4C). Additionally, image location did not affect LTS with the possible exception of 5 and 5+ classes grown in the field where the First image showed a higher LTS than Middle and Last images (Fig. 4D). These results are in line with those presented in the HairNet study [8] and suggest that the First image of L3 is a robust compromise between accuracy and higher throughput.

To investigate whether the LTS dip in GHS 4 was due to seasonal factors the CotLeaf-1 dataset was complemented with CotLeaf-2, a new dataset collected over the two following seasons (21–22, Y3, and 22–23, Y4; Table 1). Based on conclusions of Fig. 4, to create this new dataset the same 27 genotypes were grown in the Field (Y3 and Y4) and the glasshouse (Y3), but only the First image on L3 was collected (Table 1). The analysis of the L3 First images in both CotLeaf-1 and CotLeaf-2 revealed that field and glasshouse environment exhibited a comparable relationship between LTS and GHS in any given year (Fig. 5). In the Glasshouse, the LTS dip at GHS 4 was obvious in Y2 (Fig. 5B), whilst in Y1 and Y3 the LTS did not significantly increase between GHS 3 and 4 (Fig. 5A and C). In the field, the LTS dip at GHS 4 was observed every year with an additional LTS dip at GHS 4+ in Y3 and Y4 (Fig. 5A–D). When all years were combined the LTS dip at GHS 4 was detected in both field and glasshouse (Fig. 5E). This observation suggests that intermediary GHS classes 3 to 4+ may not reflect an ordinal increase in LTS.

To investigate the cause of this discrepancy, the same data was investigated at the genotype level (Fig. 6). Because Y1 only had 10 genotypes per environment there were very few genotypes with the same GHS class. Notably, that year the two genotypes with GHS of 4+ (blue and gray) showed radically different LTS values in either environment (Fig. 6A). This was also observed in Y2, Y3, and when all three years were combined (Fig. 6B to E). Interestingly, genotypes with a GHS of 4/4+ also exhibited high LTS variations, whilst those with a GHS between 3 and 4 showed LTS variation within a relatively narrow range.

### HairNet2 allows to redefine leaf hairiness rankings

Based on these observations, a new LTS-based genotype order was proposed for the glasshouse (Fig. 7A) and the field (Fig. 7B), respectively. These new ordering of genotypes were fairly similar between both environments and suggest that the correlations between a low LTS and a low GHS (1–2), and between a high LTS and a high GHS (5, 5+) are very strong as these genotypes were located at both extremes of the LTS-based rankings. However, the LTS-based order for genotypes with intermediate GHS values (3 to 4+) was significantly different to its GHS-based counterpart. For example, in the LTS-based rankings gray (GHS 4+) was in the lower half and ebony (GHS 3/4) was in the top third in either environment (Coloured arrows in (Fig. 7A and B). Because the values used in Fig. 7 were average LTS for each given genotype, the distribution of LTS values withing the 10 genotypes common to Y1, Y2 and Y3 was further investigated and showed acceptable variations, suggesting that LTS values are robust across years within a given environment (Fig. 8).

The discrepancy between GHS and LTS may be explained by a few factors. Firstly, breeders typically discard plants with a GHS lower than 3 or higher than 4+, meaning that for a human these are the important boundaries to learn to perceive well. Secondly, it is easier for a human to identify extremes than it is to subtly rank intermediate phenotypes - especially when these subtleties are currently not exploited within breeding programs. Thirdly, the GHS is attributed to a genotype based on a ’look and feel’ observation made at the macroscale, which is likely to be an integration of a number of factors. Conversely, the LTS is determined based on microscopy images. At the macroscale for example, the tactile feel of a leaf or its ability to reflect sunlight could be differently influenced by varying combinations of ’length x number’ of trichomes which could result in an identical LTS at the microscale. It is also possible that some of aspects of leaf hairiness integrated into a GHS at the macroscale could be missing in the data captured at the microscale, especially if it is located on the leaf edges.

### HairNet2 predictions correlate with human expert image rankings

In order to compare the performance of HairNet2 and human experts on leaf images, a new dataset called CotLeaf-X was created. For this dataset, two phenotypically diverse cotton populations were used: population A (relatively low hairiness) and population B (higher hairiness). First images were captured for both populations, and additional Blade images were captured for population A (Table 1 and Figs. 1 and 9A–C). All these images were ranked on arbitrary ordinal scales by experts (0 to 5 for population A, and 2 to 5.5 for population B).

Interestingly, HairNet2 was able to efficiently segment trichomes on the blade and on the edge of a leaf (Fig. 9C). This is significant because both scenarios were not present in the dataset used to train the model and it demonstrates the flexibility of HairNet2 to work well in slightly adjacent scenarios. In all three subsets (pop. A First, pop. A Blade, and pop. B First), LTS predictions from HairNet2 increased with human scores (Fig. 9D–G). This shows that unlike the partial correlation between GHS and LTS observed at the genotype level, HairNet2 and Human expert image predictions correlated strongly across hairiness scales.

The observation that the hairiest images in population A showed a higher LTS for First images than for Blade images may be explained by a number of phenomenons. One possibility is that in hairy plants the midvein area of the First image is more hairy than the Blade area, whilst in less hairy plants the difference between these two areas is minimal. Alternatively, it is possible that this discrepancy is caused by the fact that the First image comes from a fixed location when the Blade could come from a range of areas on the leaf blade, including leaf edges where only part of the image encodes plant tissue (Additional file 1).

## Conclusion

Leaf hairiness is an important cotton trait which is currently measured qualitatively by humans or with the deep-learning classifying model HairNet [8]. In this study, a number of new image datasets were created which are available at [link to be added after acceptance] and will help the broader community build digital tools to assist in the development of better crops. These datasets were used to develop and validate HairNet2, a quantitative deep-learning model able to efficiently segment and quantify leaf hairiness from images of leaf midveins, blades or edges. The output of HairNet2, Leaf Trichome Hairiness (LTS), showed that similarly to the results obtained with HairNet, leaf identity (L3/L4) and image position (First, Middle, Last) did not significantly affect genotype ranking, although LTS absolute values were different between L3 and L4. Converging with the HairNet study, growth environment and different years shows slight variations in LTS absolute values but the trends were conserved across conditions. When looking at genotypes in more details, assessments of glabrous (GHS 1–2) and pilose (GHS 5–5+) genotypes was consistent between GHS and LTS. However, a new LTS-based ranking was suggested for genotypes with intermediate GHS (3–4+). Finally, the performance of HairNet2 was shown to correlate with visual scoring of images by human experts. Overall, this study demonstrates that HairNet2 is a robust quantitative model which creates new opportunities to revisit the complex genetics which underpin leaf hairiness. In particular, it will enable the selection of plants with specific leaf hairiness characteristics which may be associated with other beneficial traits.

### Supplementary Information

**Additional file 1: Figure S1.** Qualitative results of HairNet2 predictions on the CotLeaf-X dataset.

## Data Availability

The AnnCoT image dataset is available at 10.25919/ekzz-r590. The CotLeaf-1 image dataset is available at 10.25919/9vqw-7453. The CotLeaf-2 image dataset is available at 10.25919/v0qb-er50. The CotLeaf-X image dataset is available at 10.25919/eqhx-1x73.
