# Supplementary figures and images for: HairNet2: deep learning to quantify cotton leaf hairiness, a complex genetic and environmental trait
Source: Plant Methods. 2024 Mar 19;20:46. doi: 10.1186/s13007-024-01149-8 (PMC10949638; doi:10.1186/s13007-024-01149-8)

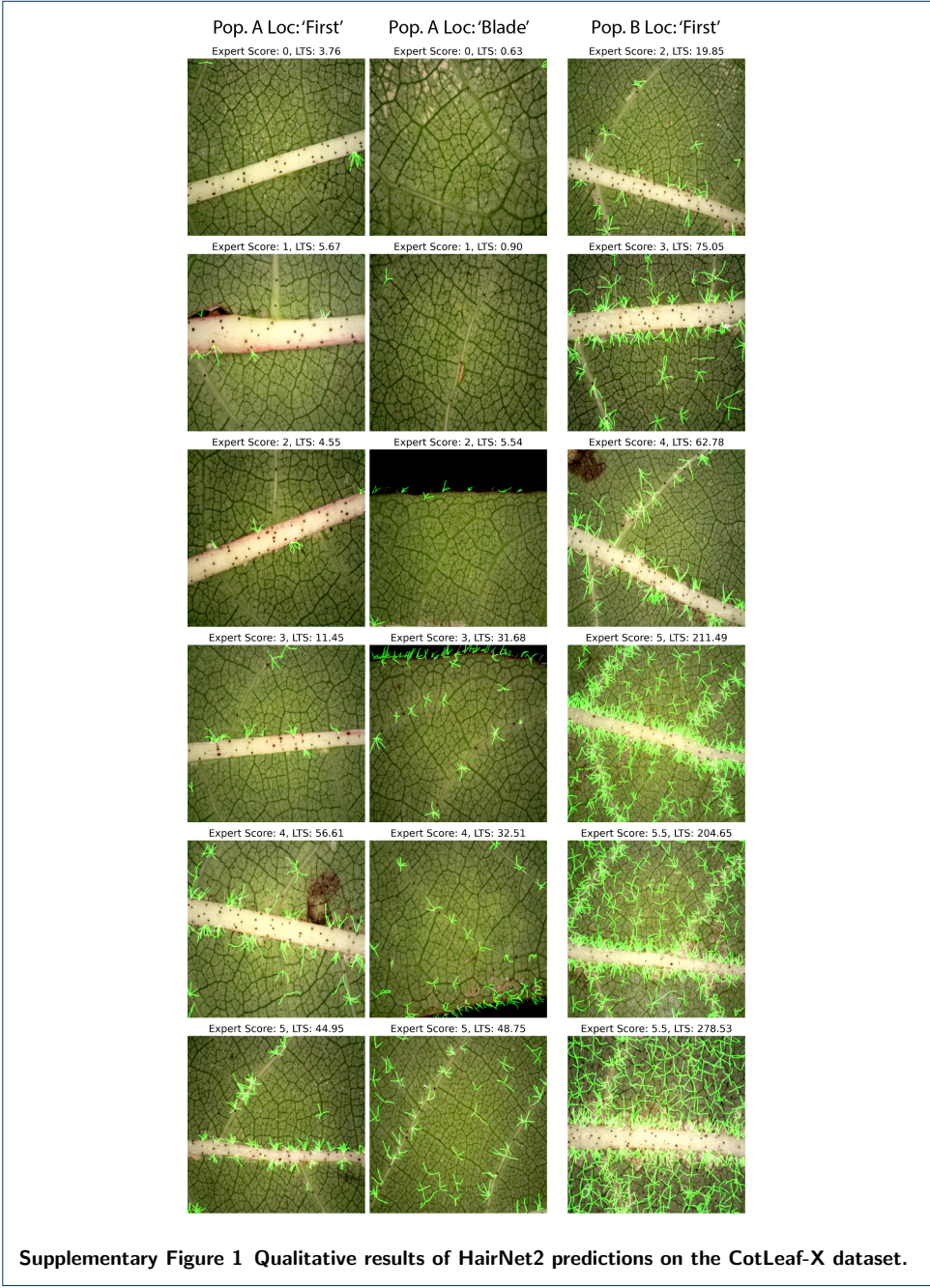

Supplement: Supplementary file 1 — Additional file 1: Figure S1. Qualitative results of HairNet2 predictions on the CotLeaf-X dataset. [file 13007_2024_1149_MOESM1_ESM.pdf]
